# Sec14-like proteins to the rescue: Improving plant performance in low-phosphate conditions

**DOI:** 10.1093/plphys/kiad265

**Published:** 2023-05-05

**Authors:** Hannah M McMillan

**Affiliations:** Assistant Features Editor, Plant Physiology, American Society of Plant Biologists, USA; Department of Biology, Duke University, Durham, NC 27708, USA

Phosphorous limitations are one of the major challenges facing modern agriculture, and improving phosphate uptake is the focus of many crop breeding programs. Although fertilizers deliver ample phosphorous to crop fields, phosphate readily interacts with cations in the soil and forms insoluble, inaccessible compounds (Schachtman et al. 1998). As a result, crops experience acute phosphorous deficiencies, which constrain normal growth and development by limiting synthesis of critical organic compounds such as nucleic acids and phospholipids (Abel et al. 2002). Current farming practices only make this issue worse. For example, intensive cropping where the whole plant is harvested removes basic material that would normally counteract soil acidification (Hao et al. 2019). Nitrogen fertilizer often directly contributes to soil acidification as ammonium is broken down to form nitrate (Hao et al. 2019). Increased acidity promotes the interaction between phosphate and metal cations in the soil, thereby restricting further plant access to phosphorous (Penn and Camberato 2019).

In response to phosphorous limitation, plants increase phosphate uptake and use efficiency by secreting compounds into the rhizosphere to release phosphorous from insoluble compounds (Raghothama and Karthikeyan 2005), upregulating expression of phosphate acquisition- and transport-related genes (Nussaume et al. 2011) and reallocating intracellular pools of phosphate through membrane remodeling and lipid recycling (Hölzl and Dörmann 2019). One way to remodel plant membranes and improve phosphate use efficiency is by replacing phosphorous-containing lipids with phosphorous-free lipids. Chloroplasts present a unique situation in plant cells because they contain significantly fewer phospholipids than other biomembranes and, therefore, are somewhat less impacted by phosphorous limitation (Hölzl and Dörmann 2019). However, plants still improve phosphate use efficiency in chloroplasts by removing phosphatidylglycerol (PG) in favor of other negatively charged lipids such as sulfoquinovosyldiacylglycerol (SQDG) (Yu and Benning 2003). Importantly, substituting PG with other anionic lipids maintains the function of organellar structures—in this case, the thylakoid membrane (Yu and Benning 2003; Hölzl and Dörmann 2019).

Despite these advances in understanding plant responses to phosphate limitation, much of the mechanism and regulation of membrane remodeling in this context remains unknown. In this issue of *Plant Physiology*, Yang et al. reveal a new role for Sec14-like proteins AtPITP7 and OsPITP6 in regulating phosphorous utilization in chloroplasts and present exciting possibilities for manipulating *OsPITP6* expression to improve growth and yield in phosphate-limiting conditions (Yang et al. 2023). In yeast, Sec14 family proteins are some of the most extensively studied lipid transfer proteins (Holič et al. 2021). Specifically, many Sec14 family proteins mediate transport of phosphatidylinositol between membrane bilayers and are also able to bind ligands such as phosphatidylcholine, sterols, squalene, phosphatidylserine, and heme (Holič et al. 2021). This function has been linked to phospholipid metabolism, membrane trafficking, and polarized membrane growth, and in mammalian cells, disruption of Sec14 family protein function contributes to a number of different diseases (Holič et al. 2021). Arabidopsis contains 32 Sec14-like proteins and rice contains 29, some of which have been shown to bind phospholipids and regulate membrane trafficking (Yang et al. 2023), similar to Sec14 family protein function in yeast and mammalian cells. Intriguingly, Arabidopsis and rice each contain only one Sec14-like protein with known localization in chloroplasts: AtPITP7 and OsPITP6 (Yang et al. 2023). Deactivating or overexpressing this gene results in significant changes in fresh shoot and root weight, primary root length, phosphate uptake activity and content, and photosynthetic capacity in both Arabidopsis and rice (Yang et al. 2023). These phenotypes are even more apparent under limited phosphate conditions, which suggests that AtPITP7 and OsPITP6 play roles in chloroplast phosphate metabolism and could be novel targets for improving crop productivity in soils with low phosphate availability (Yang et al. 2023).

**Figure 1.**
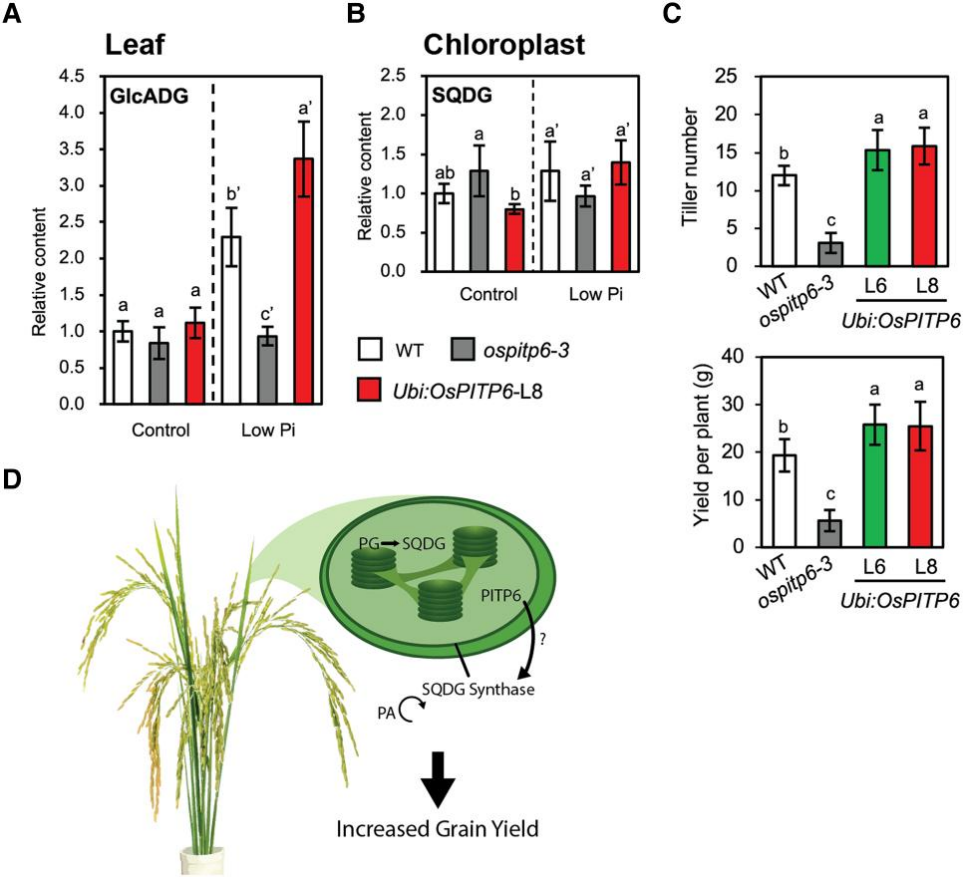
Phospholipid and glycolipid contents of the leaves and chloroplasts and agronomic traits of wild-type, *ospitp6-3* (reduced *OsPITP6* expression), and *UBI:OsPITP6* (overexpression of *OsPITP6*) plants. **A**) GlcADG content in whole leaf samples. **B**) SQDG content of chloroplasts of 4w-old wild-type (white), *ospitp6-3* (grey), and *Ubi:OsPITP6* (L8) (red) seedlings that were grown in 0.5 × Yoshida nutrient solution for 2 wk and then in the control or low Pi nutrient solution for 2 wk. **C**) Tiller number and yield per plant of 150-d-old wild-type, *ospitp6-3*, and *Ubi:OsPITP6* (L6 and L8) plants grown in soil. **D**) Model summarizing some of the findings from this study. Data represent mean ± SD of 5 biological replicates, and different letters above bars indicate statistically significant differences (*P* < 0.05; Tukey's multiple comparison test). Panels **A–C** and figure legend from Yang et al. (2023).

To determine the mechanism by which OsPITP6 mediates phosphate utilization in chloroplasts, Yang et al. examined the lipid profiles of mutants with inactive or overexpressed *OsPITP6* in normal and phosphate-limited conditions. In Arabidopsis, AtPITP7 binds phospholipids, including phosphatidylinositol and phosphatidic acid (Hertle et al. 2020). Under phosphate deficiency, plants decrease phospholipid content and increase glycolipid content (Hölzl and Dörmann 2019). In chloroplasts, where PG is the major phospholipid class, OsPITP6 may facilitate replacement under limited phosphate conditions with SQDG or glucuronosyldiacylglycerol (GlcADG), both anionic lipids that do not contain phosphorous. Indeed, Yang et al. show that the profiles of several lipid classes significantly change when *OsPITP6* expression is reduced or overexpressed in both normal and phosphate-limited conditions (Yang et al. 2023). The most striking change occurs with GlcADG and SQDG levels in the overexpression line (Fig. 1, A and B). Under normal phosphate conditions, GlcADG levels in whole leaf samples are unchanged by *OsPITP6* expression (Fig. 1A). In phosphate-limited conditions, GlcADG levels increase in wild-type plants, which could be a reflection of optimized phosphate-use efficiency, and increase even more when *OsPITP6* is overexpressed, which could be a reflection of overactive phospholipid substitution or, perhaps, improved performance under phosphate deficiency (Fig. 1A). The *OsPITP6* mutant, which is unable to produce OsPITP6, does not show a change in GlcADG levels (Fig. 1A). Similarly in chloroplasts, SQDG levels are increased further in the overexpression line than in wild type under phosphate-limited conditions, suggesting that OsPITP6 improves phosphate use efficiency by facilitating the replacement of PG with SQDG (Fig. 1B). Changes to overall lipid profiles are also reflected in PCA plots, which show greater separation of samples from the *OsPITP6* overexpression line than wild type under phosphate-limited conditions in chloroplasts, particularly along PC2 (Yang et al. 2023).

Improved phosphate utilization efficiency is particularly exciting in light of agronomically important traits that appear to be enhanced in the *OsPITP6* overexpression line (Yang et al. 2023). Rice plants overexpressing *OsPITP6* have increased root length and weight as well as increased tiller number and grain yield per plant (Fig. 1C). Importantly, Yang et al. show that Arabidopsis AtPITIP7-associated traits are dependent on the chloroplastic function of AtPITP7 and that each of 8 tested plant species has only 1 chloroplastic Sec14-like protein (Yang et al. 2023). Further, varied *AtPITP7* activity among Arabidopsis accessions is linked to promoter function, influencing downstream AtPITP7 ability to improve plant performance in phosphate-limited conditions (Yang et al. 2023). Together, these results suggest that chloroplast Sec14-like protein function could be manipulated in a wide range of crop species to increase yield in low-phosphate soils with the added benefit that Sec14-like protein genes and promoters could be used as selection markers in conventional breeding programs.

In this study, Yang et al. identify a previously overlooked target, chloroplast Sec14-like proteins, that improves phosphate use efficiency under low-phosphate conditions via lipid remodeling (Fig. 1D). Not only do the data provide important mechanistic insight into the function of Sec14-like proteins in chloroplasts, but they also show that manipulating Sec14-like function and phosphate use efficiency in general could have a tremendous impact on improving crop yield under normal and phosphate-limited conditions. Importantly, Yang et al. provide evidence that these phenomena could be expanded to a wide range of economically important crop species.
